# Does bacterial colonization influence ureteral stent-associated morbidity? A prospective study

**DOI:** 10.1080/2090598X.2022.2164124

**Published:** 2023-01-02

**Authors:** Mohamed Samir, Mahmoud Ahmed Mahmoud, Ahmed Tawfick

**Affiliations:** Urology, Ain Shams University Hospitals, Cairo, Egypt

**Keywords:** Bacterial colonization, ureteral stents, urinary symptoms, morbidity of DJ

## Abstract

**Objective:**

to evaluate the effect of bacterial colonization on ureteral stent-associated morbidity.

**Methods:**

This was a prospective study that took place between February 2019 and March 2022. We examined one hundred fifteen patients for ureteric stents application. On the same day of stent removal, the Arabic version of Ureteral Stent Symptoms Questionnaire (USSQ) was used to assess stent-associated morbidity. The stent-associated morbidity and the specificity and sensitivity of culture in the stent and midstream urine were recorded.

**Results:**

In 15.6% of the patients stent colonization was positive; E. coli was the most common isolated organism. There was no statistically significant difference between sex, age, irrigation fluid volume and duration of operation for stent colonization. However, stent indwelling time was significantly higher in patients with stents with positive cultures. In the colonized stents, there was a statistically significant difference with regards to the total score of USSQ, pain, urinary symptoms, work performance and additional problems of USSQ. Meanwhile, there was no statistically significant difference in the general health and sexual matter.

**Conclusions:**

stent colonization may be a contributing factor in stent-related morbidity. Stent bacterial colonization increases with the time of stent retention. Stent cultures are not needed as the same microorganisms are detected in urine cultures.

## Introduction

Ureteral stents have become an integral part of urological operations. Nowadays, ureteral stent application is the most frequently used procedure to relieve ureteric obstructions [1].

Ureteral stents are associated with clear side effects such as storage symptoms (nocturia, frequency, urgency and urgency urinary incontinence) as well as loin pain and hematuria [2]. Storage symptoms were reported in 78% of patients, and pain affecting daily practice in more than 80% [3].

The pathophysiology of stent-associated morbidity is unclear. It may be caused by the mechanical irritation of trigonal bladder nerves and urothelium [4]. Others have reported that there is a causal link between bacterial colonization, stent biofilm and stent-related symptoms [5].

Ureteral stents biofilm is caused by single bacterium adherence and multiplication leading to the formation of base film, then surface film [6]. Stents biofilms induce the production of various substances by urothelial cells, such as chemokines, cathelicidin, pro-inflammatory cytokines and nitric oxide. This hypothesis seems reasonable for it can lead to local inflammation, which stimulates the afferent nerves and might result in LUTS and pain [7].

In the literature, there is limited vivo data about the correlation between stent-associated morbidity and bacterial colonization. So, in our study the 1ry end point was to investigate the correlation between stent-associated morbidity and bacterial colonization. The 2ry end point was to determine the incidence rate of colonization on ureteral stents and the antibiotic sensitivity patterns of isolated microorganisms.

## Patients and method

From February 2019 to March 2022, this study was done prospectively at a single tertiary care centre. Approval of our institution’s Research Ethics Committee was obtained before the beginning of the study and all patients signed a written informed consent before sharing in our study.

The study included patients who were admitted to our hospital for double J ureteral stent application for different reasons. Exclusion criteria were patients with active UTI, stent migration, renal impairment, diabetes mellitus and immunosuppression. These patients were excluded because they are generally more susceptible to infection. All patients were evaluated by history taking and underwent general examination and baseline investigations as serum creatinine, urine analysis, complete blood count (CBC), plain x-ray of kidney, ureter and bladder (KUB), ultrasonography, non-enhanced spiral CT and CT with contrast if indicated.

Sample size was determined using the PASS program, type-1 error (α) was fixed at 0.05 and the confidence interval width at 0.1. Results from Ozgur et al. showed that bacterial colonization was present in 7.6% of the ureteric stents. So, calculation according this value resulted in a sample size of 108 cases [8].

**Chart 1.**
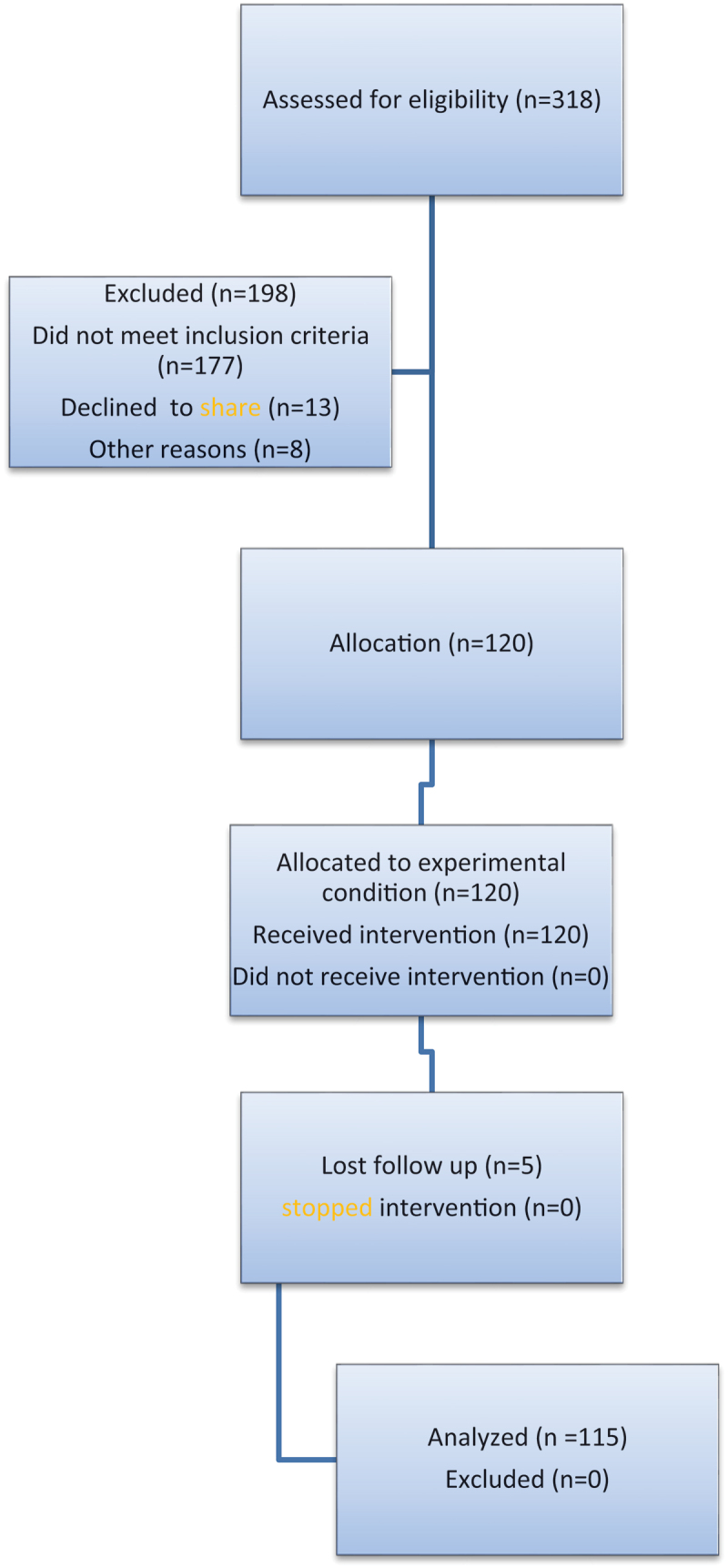
CONSORT flow chart.

Three hundred eighteen patients with ureteral stents who were eligible to be involved in the study were evaluated. One hundred ninety eight patients were excluded for different reasons as shown in chart 1.

All patients were prescribed antibiotic prophylaxis at the time of internal double pigtail ureteral stent application but not before stent removal. The stents were placed while the patients were under general or spinal anesthesia. In the lithotomy position, the stents were applied using a 22 Fr cystoscope under fluoroscopic guidance. A urethral catheter was then inserted for a whole day. All the used stents were of the same diameter, 6Fr, and made of polyurethane material (Microvasive Boston Scientific Corporation, France). The adequate length of the stent was measured using Pilcher and Patel formula according to the height of the patient [9]. The proximal coil was inserted in the renal pelvis and the distal coil was left just projected behind the vesicoureteric junction. The stents were inserted for different time durations according to the indication. The stents were removed using flexible cystoscopy in a sterile manner. Patients full filled the Arabic version of USSQ on the day of stent removal [10].

## Bacterial isolation

A midstream urine sample was collected before the application of the stent to exclude any active UTI and on the same day of stent removal. The distal 3 cm of the stent located in the bladder was cut in an aseptic manner after the stent was removed and placed in a sterile test tube and tested for colonization. Urine samples were sent for culture on eosin methylene blue agar and blood agar and examined 48 hours later. Positive cultures were defined as the growth of 100,000 colony-forming units (cfu)/ml. All isolated microorganisms were tested for their sensitivity to a panel of 19 antibiotics.

The antibiotic efficacy was calculated by determining the diameter of the zones of inhibition. Bacterial strains were classified as susceptible (S), intermediate (I), or resistant (R) according to the diameter of the inhibition zone [11].

The following data was recorded and analyzed: patient age, sex, site, previous urologic operations, indication for internal double pigtail ureteral stent application, intraoperative irrigation fluid volume during stent placement, duration of operation, stent retention time, the morbidity of the stent which was assessed by the Arabic version of USSQ, and the sensitivity and specificity of culture in the stent and midstream urine on the same day of stent removal.

## Statistical analysis

Kolmogorov–Smirnov’s test was used to assess the normal distribution of continuous variables. All results were presented as mean and SD values or as median and interquartile range according to the data distribution. Categorical results were presented as numbers of cases and percentages. Continuous variables were compared using student’s t-test or the Mann–Whitney U-test, according to the data distribution. Categorical variables were compared using chi-square test, Fisher’s exact test or Monte Carlo test depending on the data. Kappa statistics was used to compute the measure of agreement between two investigational methods: a Kappa score of over 0.75 is excellent; a score 0.40 to 0.75 is fair to good; and below 0.40 is poor. All statistical calculations were done using SPSS version 20 for Windows (SPSS Inc, Chicago, IL, USA).

## Results

Out of a total of 115 patients who underwent stent insertion and were included in the study, 96 (83.5%) were male and 19 females (16.5%). The mean age of the patients was 38.27 ± 11.87 years (ranging from 18 to 72). The indications of stent application were ureteroscopic stone extraction/lithotripsy (38.3%), prophylactic before ESWL (34.8%), after PNL (14.8%), hydronephrosis due to ureteric stricture (6.1%) and hydronephrosis due to malignancy (6.1%).

The mean irrigation fluid volume was 5.44 ± 3.87 L. The mean stent retention time was 39.74 ± 24.72 days (ranging from 14 to 110).

With regards to the pre-operative data, there was no statistically significant difference between age (P = 0.13), sex (P = 0.73), indication (p = 0.41) and previous urologic operations (p = 0.823) for stent colonization. Table 1.

**Table 1.** Comparison between patients with and without stent colonization in preoperative data.

		Stent culture result				P
		Positive		Negative		
		Mean	±SD	Mean	±SD	
Age		34.39	8.24	38.99	12.32	0.131
Sex	Male	16	16.7%	80	83.3%	0.733
	Female	2	10.5%	17	89.5%	
Indication	Hydronephrosis due to ureteric stricture	2	11.8%	15	88.2%	0.41
	Pre SWL	8	20.0%	32	80.0%	
	post PNL	2	28.6%	5	71.4%	
	post URS	4	9.1%	40	90.9%	
	Hydronephrosis due to malignant obstruction	2	28.6%	5	71.4%	
Previous urologic operations	No	12	16.2%	62	83.8%	0.823
	Yes	6	14.6%	35	85.4%	

Regarding the perioperative data, there was no significant difference between negative and positive stent colonization patients in the duration of operation and irrigation fluid volume (32.69 ± 19.68 and 5.51 ± 4.00 vs 29.94 ± 17.50 and 5.06 ± 3.13). On the other hand, the stent retention time was significantly higher in positive stent cultures patients. Table 2.

**Table 2.** Comparison between negative and positive stent culture cases as regard perioperative data.

	Stent culture result										
	Negative					Positive					
	Mean	±SD	Median	IQR		Mean	±SD	Median	IQR		P
Duration of operation (minute)	32.69	19.68	22.0	17.0	48.0	29.94	17.50	24.0	19.0	39.0	0.552
Irrigation fluid volume(liters)	5.51	4.00	5.0	2.0	6.0	5.06	3.13	4.0	3.0	6.0	0.559
Stent retention time (days)	36.51	22.35	33.0	14.0	59.0	57.17	29.93	67.0	42.0	79.0	**0.003**

In our study group, stent bacterial colonization was detected in 18 of 115 patients (15.6%). Five out of 18 patients (27.7%) were both stent and urinary culture positive and one out of 115 patients (0.86%) was urinary culture positive only (Beta hemolytic strep). Stent and urinary cultures showed the same bacterial isolates. Urine culture had a 27.8% sensitivity and a 99% specificity for detection of stents bacterial colonization with a 83.3% and 88% positive and negative predictive value respectively. The overall accuracy of the urinary culture is 89.14%. The distribution of microorganisms found in the stents’ cultures can be found in Table 3 and the antibiotic susceptibility of the bacterial colonies identified from the stents. Table 4.

**Table 3.** Pathogens colonizing the stent.

Stent culture result	Negative	97	84.3
	E.coli	6	5.2
	Staph aureus	2	1.7
	Coag negative staph	2	1.7
	Staph saprophyticus	1	.9
	Staph epidermidis	1	.9
	Pseudomonas	1	.9
	Proteus	1	.9
	Klebsiella and e. coli	1	.9
	Klebsiella	1	.9
	Enterococcus	1	.9
	Enterobacter	1	.9

**Table 4.** Antibiotic sensitivity pattern of stent bacterial isolates.

	N	%
Nitrofurantoin	15	83.33
Vancomycin	14	77.78
Linezolid	10	55.56
Amikacin	10	55.56
Meropenem	10	55.56
Gentamicin	9	50.00
Levofloxacin	9	50.00
Ceftriaxone	6	33.33
Ceftazidime	6	33.33
Ciprofloxacin	5	27.78
Doxycycline	5	27.78
Trimethoprim and sulphamethaxole	5	27.78
Tetracyclin	4	22.22
Imipenem	4	22.22
Piperacillin	3	16.67
Amoxycillin and clavulanic acid	3	16.67
Ampicillin and sulbactam	2	11.11
Erythromycin	0	0.00
Moxifloxacin	0	0.00

Regarding the morbidity of the stent in the presence or absence of colonization, there was a highly statistically significant difference with regards to the total score of USSQ, urinary symptoms and pain. Additionally, there was a statistically significant difference in the work performance and additional problem of USSQ. On the other hand, there was no statistically significant difference in the areas of general health and sexual matter. Table 5.

**Table 5.** Morbidity of the DJ.

	DJ culture				p
	Negative		Positive		
	Mean	±SD	Mean	±SD	
Urinary symptoms	25.30	10.40	38.39	8.65	0.0001
Pain	18.71	6.01	26.17	4.99	0.000
General health	17.94	3.58	18.11	4.10	0.854
Work performance	28.84	6.97	33.22	7.67	0.017
Sexual matter	5.44	2.27	6.61	3.24	0.158
Additional problem	11.58	3.85	14.17	3.79	0.014
Total USSQ	107.80	14.76	133.94	13.97	0.0001

## Discussion

Ureteral stents were first described in 1967 and since then, the indications for stent insertion have increased. During the last few years, DJ ureteric stents have become an integral part of urological treatment and one of the most used ureteral stents [12,13].

Ureteral stents are made of synthetic biomaterials with suitable surfaces for bacterial colonization and formation of polysaccharides biofilm. Beneath this biofilm, bacterial microcolonies are formed [14].

There is a limited information on the relationship between ureteral stent morbidity and stent colonization. Some studies have reported that biofilms and bacteria stimulate the secretion of antibacterial substances and cytokines leading to local inflammation; these were documented in an animal and in vitro study [6,7,15]. Bonkat et al. were the first to correlate bacterial stent colonization with worsening of LUTS. However, there is no validated questionnaire on this study [5]. The same data was obtained from our study where a statistically significant difference was found between the presence and absence of colonization regarding the total score of USSQ, urinary symptoms, pain, work performance and additional problem of USSQ. On the other hand, Betschart et al. reported that no statistically significant relationship was found between the presence or absence of bacteriuria and stent-related symptoms evaluated as the USSQ total score, or the tested USSQ sub scores [3]. This may be since patients were instructed to take analgesics and alpha blockers and antibiotic if suspected that UTI might influence the degree of symptoms.

In the literature, there are different rates of infection in ureteral stents, which range between 9.3%–100% [16]. In our study, stent colonization and bacteriuria were low, between 15.6% and 3% respectively, as we excluded immunocompromised patients. The same results were obtained by Ozgur et al., Yeniyol et al. and Özden et al., who detected urinary tract infection in 15%, 16%, and 18% of patients with DJ respectively [8,17,18]. Others reported a higher rate of stent colonization and bacteriuria. In Riedl et al., the incidence rate of stent colonization and bacteriuria was 100% and 45% [19]. In Farsi et al., it was reported at 68% and 30% [20]; 90% and 27% in Reid et al. [21]; and 31% and 13% in Lifshitz et al. [22] Akay et al. reported 24.2% and 22.5% [23]. Furthermore, Paick et al., Shabeena et al. and Kehinde et al. reported stent colonization rates of 44%, 47.2 and 42%, respectively [24–26].

UTI is more common in females due to their short urethra and because the vaginal flora is frequently infected by enteric floras [14]. In our study, we didn’t find any relation between sex and stent colonization. This is also reported by Ozgur et al. and Özden et al. [8,14] Akay et al. and Al-Ghazo et al. reported higher colonization rates in females but it was not statistically significant [23,27]. On the other hand, Bonkat et al. in 2011 and 2013, and Kehinde et al., reported a higher rate of stent colonization and bacteriuria in females [5,13,28].

UTI can increase with age, but we didn’t find any relation between age and stent colonization [29]. This is also reported by Ozgur et al. [8] Moreover, Akay et al. reported that UTI is more common in patients older than 40 years, which was not statistically significant [23]. At the same time, Al-Ghazo et al. found that no statistically significant effect of age on bacteriuria or stent colonization (P = 0.48) [27].

With increasing stent indwelling time, biofilms have been shown to occur more frequently and in increasing amounts [30]. In our study, there is a high significant correlation between stent retention time and stent colonization. This is also reported by many studies such as that of Ozgur et al., who reported the rate of colonization at 2.2% and 25% when stent retention time was less than 4 weeks and more than 6 weeks and Shabeena et al. who reported no bacterial colonization within the first 2 weeks of stent placement. However, colonization increases to 66.7% and 81.3% of the stents when placed for 60–90 days and 90–120 days, respectively [8,25]. Kehinde et al. found a colonization rate of 4.2% for stents removed before 30 days and 34% for stents removed after 90 days while Farsi et al. reported that colonization was 58.6% for stents left for more than 1 month vs. 75.1% for those left for over 3 months [13,20]. Moreover, Al-Ghazo et al. and Paick reported that bacteriuria and stent colonization have a statically significant relation with the durations of stent insertion [24,27]. On the other hand, Akay et al. concluded that the colonization rate on the stent increased with the duration of the stent placement, but the difference was not statistically significant [23].

In our study, the most common isolated organism is E. coli. This is also reported by others as Al-Ghazo et al., Akay et al., Shabeena et al., and Özden et al. [18,23,25,27] Contrarily, Paick et al. found different organisms; the most commonly isolated pathogens was Staphylococcus, which may be a result of urethral contamination [24]. Furthermore, Farsi et al. and Shabeena et al. found that the most common isolated organisms were Gram-positive cocci (77%) and (55%) [20,25].

The study has some limitations as there has been no assessment of total biofilm mass, no specification of biofilm components and lack of influence of colonization in long-term ureteral stenting.

## Conclusion

Ureteral stent morbidity may be related to stent colonization. Indwelling time is a risk factor for stent colonization; age and sex do not affect colonization.

## Abbreviations

USSQ Ureteral Stent Symptoms Questionnaire, LUTS lower urinary symptoms, UTI urinary tract infection, CBC complete blood count, KUB plain x-ray of kidney, ureter and bladder, CT computerized tomography, DJ double J, Fr French, cfu colony-forming units, ESWL extracorporeal shock wave lithotripsy, PNL percutaneous nephrolithotomy.
